# Machine Learning for Intraoperative Bleeding Prediction in Patients Undergoing Surgery: Scoping Review

**DOI:** 10.2196/80930

**Published:** 2026-06-10

**Authors:** Shiqiong Yan, Ping Zhang, Wanwan Qiao, Sijia Xie, Huan Hu, Yi Gao, Linli Xie, Jie Jing

**Affiliations:** 1 Department of Nursing, Sichuan Provincial People’s Hospital, University of Electronic Science and Technology of China, No. 32, West Section 2, First Ring Road, Qingyang District, Chengdu, 610072, China, 86 028-87393999, 86 028-87393999; 2 Department of nursing, Chengdu University of Traditional Chinese Medicine, Chengdu, China

**Keywords:** intraoperative bleeding, machine learning, scoping review, clinical decision support, predictive models

## Abstract

**Background:**

Intraoperative bleeding is a critical event that impacts surgical safety and patient outcomes. Machine learning (ML) has demonstrated potential in prediction tasks, yet its methodological rigor and clinical translation face challenges.

**Objective:**

This scoping review aims to systematically synthesize the current state of development, performance, and validation of ML models for predicting intraoperative bleeding, and to identify key barriers to their clinical implementation.

**Methods:**

Following the PRISMA-ScR (Preferred Reporting Items for Systematic Reviews and Meta-Analyses extension for Scoping Reviews) guidelines, we systematically searched 7 databases (PubMed, Web of Science, Embase, CINAHL, CNKI [China National Knowledge Infrastructure], Wanfang, and VIP [China Science and Technology Journal Database]) from their inception to April 2025. Moreover, 2 reviewers (SY and PZ) independently screened studies, extracted data using the Checklist for Critical Appraisal and Data Extraction for Systematic Reviews of Prediction Modeling Studies (CHARMS), and assessed the risk of bias using the Prediction Model Risk Of Bias Assessment Tool (PROBAST). A narrative synthesis was used for data analysis.

**Results:**

Out of 2651 screened records, 23 studies were included (sample sizes ranging from 48 to 48,543). Tree-based ensemble models (eg, random forests and extreme gradient boosting) were the most frequently used (16/23, 70%), followed by logistic regression (13/23, 57%), and deep learning (11/23, 48%). Model discrimination varied widely (mean area under the curve [AUC] 0.82, SD 0.08, range 0.63‐0.93). Integration of multimodal data (electronic health records+imaging) was associated with higher performance. However, model validation was often inadequate; only 6 studies (6/23, 26%) performed external validation, and performance often declined (eg, AUC decreased from 0.85 to 0.63 in 1 study). Reporting exhibited selective bias; AUC was commonly reported (19/23, 83%), whereas key classification metrics, such as calibration (10/23, 43%) and precision (4/23, 17%), were often omitted. PROBAST assessment indicated a high risk of bias in all included studies (23/23, 100%).

**Conclusions:**

While ML models demonstrate technical promise for predicting intraoperative bleeding, our PROBAST assessment revealed a universally high risk of bias across all included studies. This fundamental methodological limitation, coupled with a severe lack of external validation and poor transparency in reporting, severely constrains the current clinical reliability of these models. Future research must prioritize prospective multicenter validation, adherence to Transparent Reporting of a Multivariable Prediction Model for Individual Prognosis or Diagnosis (TRIPOD) reporting guidelines, and enhanced model interpretability to bridge the gap toward clinical utility.

## Introduction

### Background

Perioperative bleeding is a significant risk factor for surgical procedures and is strongly linked to increased patient mortality, higher rates of postoperative complications, and excessive use of health care resources [1]. Intraoperative bleeding control effectiveness directly impacts both surgical safety and patient outcomes [2]. Excessive blood loss compromises the surgical field. It prolongs the duration of surgery [3] while also increasing the risk of severe adverse events, such as myocardial infarction and acute kidney injury [4]. While patient blood management strategies focus on optimizing preoperative risk assessment, facilitating real-time intraoperative interventions, and guiding postoperative transfusion decisions through accurate predictions of blood loss [5], current clinical practice still struggles with the reliability of predictive tools.

Intraoperative blood loss, quantified as estimated blood loss, is a fundamental quantitative metric in perioperative management, providing critical evidence to guide fluid resuscitation strategies, transfusion decisions, and the prevention and control of postoperative complications. Consequently, monitoring accuracy is regarded as a quality standard for perioperative care [6]. However, current clinical assessment methods exhibit dual limitations—subjective assessment techniques (eg, visual estimation of soaked gauze or suction canister volume) are susceptible to operator experience, resulting in high error rates, and calculation-based methods (relying on material weight differences) struggle to capture the dynamic blood loss process in real time [2]. Such inaccuracies can lead to erroneous transfusion decisions. Research has confirmed that inappropriate transfusion is an independent risk factor for postoperative infection and organ dysfunction [7]. Although existing risk assessment tools (the surgical blood loss score) are widely used [5,8,9], their inherent weaknesses, namely heterogeneous scoring criteria and a lag in advances in surgical techniques, are becoming increasingly apparent.

Although traditional prediction models (such as logistic regression) are widely used, they are constrained by linear assumptions and fail to effectively capture complex nonlinear interactions and multicollinearity among variables. Evidence suggests that predictive accuracy based on clinical experience is significantly lower than that achieved by machine learning (ML) methods [10]. With the advancement of hospital information platforms, vast amounts of high-dimensional, heterogeneous clinical data have been accumulated. Due to its unique advantages in processing such data and identifying nonlinear patterns [11], ML has rapidly emerged as a research hotspot in the field of intraoperative bleeding prediction. However, the existing body of research evidence exhibits significant fragmentation. Studies predominantly concentrate on single surgical procedures (eg, cesarean section [12] and spinal surgery [13]), resulting in a scarcity of cross-scenario algorithm comparisons; equally important, the methodological quality and validation rigor of these models are highly variable and often inadequate. Methodological limitations (such as inconsistent data preprocessing and the absence of standardized validation frameworks) have yet to be systematically evaluated and standardized. More critically, the clinical translation pathway is severely hindered by inadequate model generalizability, largely due to a pervasive lack of robust external validation. This fragmented landscape and lack of comprehensive evaluation, coupled with unaddressed methodological concerns, critically impede the understanding of ML’s actual value and the identification of optimal implementation pathways for intraoperative bleeding prediction, necessitating the urgent integration and assessment of these methodologies through systematic approaches.

### Research Objective

Therefore, based on the PRISMA-Scr (Preferred Reporting Items for Systematic Reviews and Meta-Analyses extension for Scoping Reviews) framework [14], this study establishes the following objectives:

1. To show how ML algorithms are used to predict bleeding during surgery in different settings;
2. To look at how ways of building and testing models (like picking features or choosing algorithms) affect their results (such as sensitivity and specificity);
3. To find the best-performing algorithms and define the criteria to judge them in specific fields; and
4. To highlight key problems that slow real-world use and suggest practical steps for future research and practice.

## Methods

### Overview

This scoping review was conducted following the methodological framework proposed by Arksey and O’Malley [15] and reported in accordance with the PRISMA-ScR guidelines [14] to ensure transparency and consistency. Given the focus on prediction models, the Checklist for Critical Appraisal and Data Extraction for Systematic Reviews of Prediction Modeling Studies (CHARMS) [16] was used to guide data extraction.

### Search Strategy

A systematic literature search was conducted on April 10, 2025. The search followed the Population, Intervention, Comparator, Outcome, and Study design (PICOS) framework. Both controlled vocabularies (eg, Medical Subject Headings for PubMed and Emtree for Embase) and free-text terms were used. Searches focused on three concepts—population (patients undergoing surgery), predictive tool (ML models), and outcome (risk of bleeding during surgery). In total, seven databases were searched—PubMed, Web of Science, Embase, CINAHL Complete, CNKI (China National Knowledge Infrastructure), Wanfang Data, and VIP (China Science and Technology Journal Database). Table 1 details search strategies for each database. Reference lists of included studies and leading journals were also manually screened.

**Table 1.** Search terms used to find studies.

Database	Hits, n	Search strategy
PubMed	86	(“Machine Learning”[Mesh] OR “Artificial Intelligence”[Mesh] OR “machine learning”[tiab] OR “deep learning”[tiab]) AND (“Surgery”[Mesh] OR “Surgical Procedures, Operative”[Mesh] OR surg[tiab] OR intraoperative[tiab]) AND (“Intraoperative Complications”[Mesh] OR “Hemorrhage”[Mesh] OR “Blood Loss, Surgical”[Mesh] OR bleed[tiab] OR “blood loss”[tiab])
Web of Science	79	TS=((“machine learning” OR “artificial intelligence”) AND (surg* OR intraoperative) AND (bleed* OR “blood loss” OR hemorrhag*))
Embase	220	(‘machine learning’/exp OR ‘artificial intelligence’/exp OR ‘machine learning’:ab,ti) AND (‘surgery’/exp OR ‘intraoperative period’/exp OR surg:ab,ti) AND (‘intraoperative bleeding’/exp OR ‘surgical blood loss’/exp OR bleed:ab,ti)
CINAHL Complete	1709	(MH “Machine Learning+” OR TI “machine learning” OR AB “artificial intelligence”) AND (MH “Surgery, Operative+” OR TI surg* OR AB intraoperative) AND (MH “Intraoperative Complications+” OR MH “Blood Loss, Surgical+” OR TI bleed* OR AB “blood loss”)
CNKI^a^	212	(SU=(‘machine learning’ OR ‘deep learning’ OR ‘artificial intelligence’)) AND (SU=(‘surgery’ OR ‘intraoperative’ OR ‘surgical procedure’)) AND (SU=(‘intraoperative bleeding’ OR ‘surgical bleeding’ OR ‘blood loss’))
Wanfang Data	331	(Subject:(“machine learning” OR “artificial intelligence”)) AND (Subject:(“surgery” OR “surgical”)) AND (Subject:(“intraoperative bleeding” OR “surgical bleeding”))
VIP^b^	12	(U=(‘machine learning’ OR ‘artificial intelligence’)) AND (U=(‘intraoperative bleeding’ OR ‘surgical blood loss’)) AND (M=(‘surgery’) OR T=(‘surgical patients’))

a CNKI: China National Knowledge Infrastructure.

b VIP: China Science and Technology Journal Database.

### Study Selection

Initial search records were imported into EndNote X9 (Clarivate). Duplicates were removed using automated and manual deduplication. Moreover, 2 reviewers (SY and PZ) independently screened titles and abstracts for relevance, recording decisions separately. For records retained after screening, both assessed full-text articles for eligibility and recorded decisions independently. Assessment was blind to ensure objectivity. Disagreements were resolved through discussion or, if needed, a third senior researcher (HH). A systematic review decision matrix (Table 2) guided the application of eligibility criteria.

**Table 2.** Eligibility criteria.

Category	Inclusion criteria	Exclusion criteria
Population	Adult patients (≥18 y) undergoing surgery	―^a^
Predictive tool	ML-based models explicitly developed to predict intraoperative bleeding risk	Models predicting only postoperative bleeding or failing to distinguish intraoperative or postoperative outcomes
Outcome reporting	Reported at least one performance metric: area under the curve (AUC), sensitivity, or specificity	―
Study design	Primary research: retrospective or prospective cohort studies, case-control studies	Conference abstracts, reviews, case reports, editorials, letters
Publication status	Full text in Chinese or English (including peer-reviewed preprints)	Non–peer-reviewed manuscripts, publications not in Chinese or English
Data source	―	Nonclinical or invalid sources: animal experiments, simulated datasets, nonhospital data

a Not applicable.

### Eligibility Criteria

Studies that did not meet the inclusion criteria were excluded during screening. Eligibility was determined using predefined criteria outlined in Table 2. The review decision matrix applied these criteria to the full texts to determine whether studies reported outcomes of intraoperative bleeding prediction.

### Data Extraction and Synthesis

Data extraction was performed independently by 2 reviewers (SY and PZ) using a standardized electronic form based on the aforementioned CHARMS checklist. The reviewers extracted data on the following: (1) study characteristics (author, year, country, design, sample size, surgical type, and data source), (2) model development (candidate and final predictors, data preprocessing, and ML algorithms), and (3) model performance and validation (validation method, performance metrics such as area under the curve [AUC], sensitivity, specificity, precision, and calibration). Any discrepancies were resolved through discussion or by consultation with a third reviewer (HH). Given methodological heterogeneity across studies, including differences in algorithms, validation strategies, and outcome reporting, a narrative synthesis was used for data analysis. The primary studies in this review reported model performance metrics (eg, AUC and sensitivity) and their CIs, not traditional hypothesis-testing *P* values for intergroup comparisons. Therefore, *P* values were neither extracted nor assessed. This approach aligns with the methodological focus of prediction model research.

### Risk of Bias and Quality Assessment

The risk of bias and applicability of the included studies were rigorously assessed using the Prediction model Risk of Bias Assessment Tool (PROBAST) [17]. PROBAST tool covers 4 domains—participants, predictors, outcome, and analysis. Furthermore, 2 reviewers (SY and PZ) independently assessed each study, with disagreements resolved by consensus or consultation with a third researcher (HH). The results of this assessment are summarized descriptively in the Results section.

### Ethical Considerations

This study did not require ethical approval. We did not study any human or animal subjects, and we did not collect personal information or sensitive data.

## Results

### Search Results

**Figure 1.**
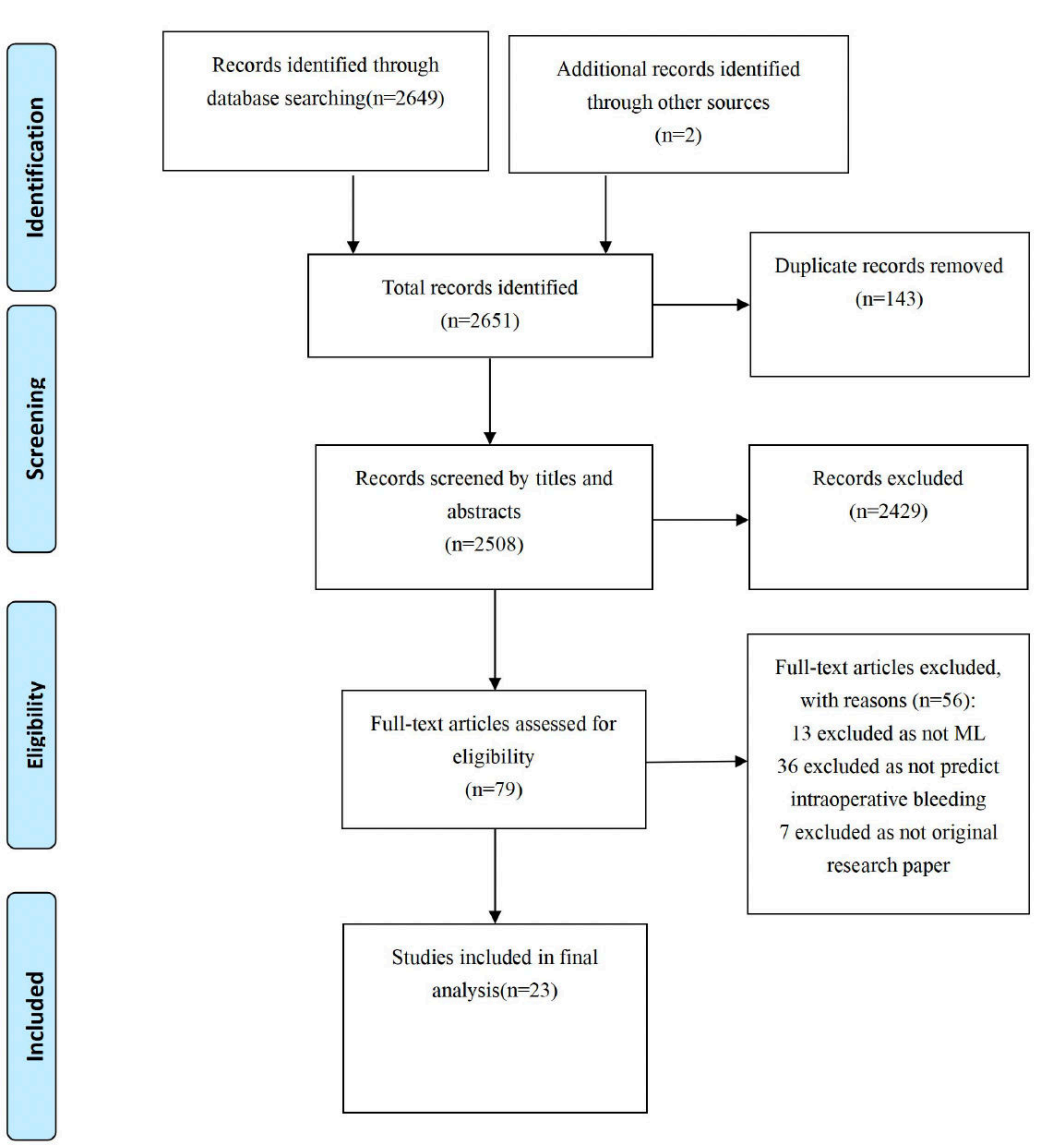
Flow diagram of the review process and the identification of studies via databases. ML: machine learning.

The systematic search initially identified 2651 records. After removing 143 duplicates, 2508 records were screened based on titles and abstracts. Of these, 2429 records were excluded. The full texts of the remaining 79 articles were assessed for eligibility, of which 56 were excluded for reasons detailed in Figure 1. Consequently, 23 studies [10,12,13,18-37] met the inclusion criteria and were included in this scoping review (Figure 1).

### Characteristics of Included Studies

The detailed characteristics of the 23 included studies [10,12,13,18-37] are presented in Table 3. The sample sizes varied widely, ranging from 48 to 48,543 cases. All studies were retrospective in design, with 17 (74%) [10,20,21,23-31,33-37] being single-center investigations. The publication years were concentrated between 2019 and 2025, and the geographical distribution was highly skewed, with studies from China dominating (17/23, 74% [12,13,20-22,24,26,27,29-37]). The main surgical contexts were obstetric procedures (10/23, 43% [10,12,18,21,27,29,32,34,36,37]), orthopedic surgery (4/23, 17% [13,19,26,31]), and hepato-biliary surgery (4/23, 17% [20,23,30,35]). Considerable heterogeneity was observed in the definitions of intraoperative major bleeding across studies, ranging from ≥200 mL to >5000 mL.

**Table 3.** Characteristics of the included studies (n=23).

Author, year	Country	Study design	Surgical type (Specific procedure)	Sample size (Development/Validation)	Data source	EBL^a^ definition
Akazawa and Hashimoto [10], 2023	Japan	Single-Center Retrospective Cohort Study	Obstetric (Cesarean section)	48	MRI^b^ + EMR^c^	≥2000 mL
Akazawa and Hashimoto [18], 2024	Japan	Multi-Center Retrospective Cohort Study	Obstetric (Cesarean section)	63 (50/13)	MRI + EMR	＞2000 mL
Chen et al [12], 2024	China	Multi-Center Retrospective Cohort Study	Obstetric (Cesarean section)	1975 (1680/295)	EMR	≥300 mL
de Reus DC et al [19], 2025	United States, Netherlands, and United Kingdom	Multi-center Retrospective Cohort Study	Orthopedic (Spinal decompression)	880	EMR	>2500 mL
Li et al [20], 2024	China	Single-Center Retrospective Study	Hepatic (Tumor resection)	406 (284/122)	EMR	≥1000 mL
Liu et al [21], 2020	China	Single-Center Retrospective Study	Obstetric (Cesarean section)	210	MRI	≥500 mL
Mo et al [22], 2023	China	Multi-center Retrospective Study	Gynecological (Hysteroscopic surgery)	200 (120/80)	EMR	≥200 mL
Park et al [23], 2022	South Korea	Single-Center Retrospective Study	Hepatic (Transplantation)	414	EMR	≥5000 mL
Shi et al [13], 2024	China	Multi-center Observational Cohort Study	Orthopedic (Spinal decompression)	276 (200/76)	EMR	≥2500 mL
Shi et al [24], 2023	China	Single-Center Retrospective Cohort Study	Multi-departmental surgeries	48,543	EMR	>200 mL
Stehrer et al [25], 2019	Austria	Single-Center Retrospective Study	Craniofacial (Orthognathic surgery)	950 (760/190)	EMR	Calculated using hemoglobin balance method
Sun et al [26], 2025	China	Single-Center Retrospective Study	Orthopedic (Lumbar fusion)	2054 (1437/617)	EMR	≥500 mL
Wang [27], 2023	China	Single-Center Retrospective Study	Obstetric (Cesarean section)	168 (117/51)	EMR	>1000 mL
Wakiya et al [28], 2021	Japan	Single-Center Retrospective Cohort Study	General (Pancreatic cancer resection)	175 (128/47)	EMR	>20% of circulating blood volume
Xu [29], 2024	China	Single-Center Retrospective Study	Obstetric (Cesarean section)	249 (149/50/50)	MRI + EMR	≥1000 mL
Xue et al [30], 2021	China	Single-Center Retrospective Study	Hepatic (Tumor resection)	665 (466/199)	EMR	≥800 mL
Yang et al [31], 2022	China	Single-Center Retrospective Study	Orthopedic (Spinal fracture)	161	EMR	Hidden blood loss (no explicit quantitative threshold)
Yang et al [32], 2023	China	Multi-center Retrospective Cohort Study	Obstetric (Cesarean section)	125 (85/40)	MRI + EMR	≥1500 mL
Yin et al [33], 2021	China	Single-Center Retrospective Study	Oncological (Pelvic/sacral tumors)	810	CT^d^ + EMR	>3000 mL
Zheng et al [34], 2024	China	Single-Center Retrospective Study	Obstetric (Cesarean section)	346 (156/68/122)	MRI + Coagulation tests + EMR	>1000 mL
Zheng et al [35], 2022	China	Single-Center Retrospective Study	Hepatic (Tumor resection)	336 (268/68)	EMR	≥300 mL
Zong et al [36], 2024	China	Single-Center Retrospective Cohort Study	Obstetric (Cesarean section)	323 (227/96)	MRI + EMR	≥1500 mL
Li [37], 2024	China	Single-Center Retrospective Case-Control Study	Obstetric (Cesarean section)	231	EMR	≥1500 mL

a EBL: estimated blood loss.

b MRI: magnetic resonance imaging.

c EMR: electronic medical record.

d CT: computed tomography.

### Technical Features and Performance of Prediction Models

All models were based on electronic health records (EHRs). A total of 8 studies (35%) [10,18,21,29,32-34,36] further integrated medical imaging data, including magnetic resonance imaging (MRI) or computed tomography, of which 7 (30%) [10,18,21,29,32,34,36] focused on predicting obstetric bleeding. In terms of algorithms, tree-based ensemble models were most frequently applied (12/23, 52% [10,12,13,23-25,27,30-32,35,37]), especially random forests (8/23, 34% [12,13,23,25,27,31,32,35]) and extreme gradient boosting (9/23, 39% [10,12,13,23,24,30,32,37]); logistic regression (13/23, 57% [12,13,18,20,23,24,26,27,30-33,37]) and deep learning (6/23, 26% [10,21,22,30,33,36]) models were also commonly used. Model discrimination performance is illustrated in Table 4. The AUC ranged from 0.63 to 0.93, with a mean of 0.82 (SD 0.08). Models incorporating multimodal data (eg, EHR+imaging) showed a performance advantage (mean AUC≈0.84, SD 0.075) over unimodal models relying solely on EHR (mean AUC≈0.82, SD 0.069). For instance, the support vector machine model by Xu [29], which fused MRI radiomic features with clinical data, achieved an AUC of 0.87.

**Table 4.** Characteristics and validation performance of ML^a^ prediction models in included studies (n=23).

Author	Predictors categories	ML algorithms	Best model	Internal validation (test set performance)	External validation performance	Validation methods
Akazawa and Hashimoto [10]	MRI^b^, laboratory parameters, demographic characteristics	Multimodal DL^c^, XGBoost^d^, VGG16^e^	Multimodal DL	AUC^f^=0.73 (95% CI 0.66‐0.80), Accuracy=0.68	Not reported	Random split (8:2), cross-validation
Akazawa and Hashimoto [18]	Radiomics features, clinical variables	LR^g^	LR	AUC=0.69 (95% CI 0.62‐0.75)	AUC=0.70 (95% CI 0.66‐0.73)	Internal: random split (7:3), external: another institution
Chen et al [12]	Clinical variables	Bayes^h^, MLP^i^, DT^j^, KNN^k^, LR, RF^l^, SVM^m^, XGBoost	Bayes	AUC=0.82 (95% CI 0.80‐0.84), Sensitivity=0.93, Specificity=0.42, *F* score=0.92	AUC=0.85 (95% CI 0.83‐0.87), Sensitivity=0.95, Specificity=0.50, *F* score=0.96	Internal validation: 10-fold cross-validation, (8:2 split), multicenter external validation
de Reus DC et al [19]	Tumor type, ECOG^n^ score, surgical procedure, preoperative platelet count	Not reported	Not reported	Not reported	AUC=0.63 (95% CI 0.58‐0.68), Sensitivity=0.74, Specificity=0.41, *F* score=0.33	Multicenter external validation
Li et al [20]	Demographic characteristics, laboratory parameters, imaging characteristics, pathological characteristics	LR	LR	AUC=0.80	Not reported	Random split (training set:test set=7:3)
Liu et al [21]	MRI	DL	VGG16	Accuracy=0.75, Sensitivity=0.73, Specificity=0.77	Not reported	5-fold cross-validation
Mo et al [22]	Clinical variables	DNN^o^	DNN	Accuracy=0.91, Sensitivity=0.89, Specificity=0.92, Precision=0.92	Not reported	Training:test=6:4
Park et al [23]	Laboratory parameters, surgical parameters, MELD^p^ score, demographic characteristics	LR, Elastic Net, SVM, RF, XGBoost, NN^q^	LR	AUROC^r^=0.84, AUPR^s^=0.82	Not reported	Training:test=7:3, feature selection via nested cross-validation
Shi et al [13]	Tumor type, ECOG score, surgical procedure, preoperative platelet count	LR, KNN, DT, XGBoost, RF, SVM	XGBoost	AUC=0.85 (95% CI 0.82‐0.87), Accuracy=0.77, Recall=0.85, *F* score=0.78, Precision=0.72	AUC=0.80(95% CI 0.77‐0.86), Accuracy=0.73, Recall=0.73, *F* score=0.73, Precision=0.73	Internal validation: random split (7:3 ratio), external validation: independent cohort
Shi et al [24]	Surgical parameters, laboratory parameters, demographic characteristics	LGB^t^, XGBoost, CatB^u^, AdaB^v^, LR, LSTM^w^, MLP	LGB	AUC=0.93, Accuracy=0.87, Sensitivity=0.8, Specificity=0.85	Not reported	Training:test =2:1, ADASYN^x^ was used to address data imbalance
Stehrer et al [25]	Surgical parameters, laboratory parameters, demographic characteristics	RF	RF	Regression performance: significant correlation between predicted and actual values; mean error 7.4 (SD 172.3) mL	Not reported	Random split (training:test=8:2), performance evaluation: correlation and mean error between predicted and actual values
Sun et al [26]	Surgical parameters, laboratory parameters, demographic characteristics	LR	LR	AUC=0.73 (95% CI 0.67‐0.79), Accuracy=0.88	Not reported	Random split (training set:test set=7:3), 5-fold cross-validation
Wang [27]	Radiomics features, clinical variables	LR, SVM, RF, SGD^y^, KNN	LR	AUC=0.83, Accuracy=0.80, Sensitivity=0.75, Specificity=0.83	Not reported	Random split (training set:test set=7:3), 5-fold cross-validation
Wakiya et al [28]	Surgical parameters, laboratory parameters, tumor markers	DT	DT	Accuracy=0.80, Sensitivity=1, Specificity=0.66	Not reported	Random split (training set:test set=3:1)
Xu [29]	Radiomics features, clinical features	SVM	SVM	AUC=0.87, Accuracy=0.85, Sensitivity=0.72, Specificity=0.89	Not reported	Random split: training:validation:test=6:2:2
Xue et al [30]	Laboratory parameters	LR, DT, XGBoost, CNN^z^, LSTM	XGBoost	AUC=0.72, Accuracy=0.87, Precision=1, Recall=0.18, *F* score=0.31	Not reported	Random split (training set:test set=7:3), 5-fold cross-validation
Yang et al [31]	Demographic characteristics, surgical parameters, laboratory parameters	XGBoost, LR, LGBM, RF, SVM	RF	AUC=0.86, Accuracy=0.78, Sensitivity=0.86, Specificity=0.81	Not reported	Random split into training and internal validation sets; 15-fold cross-validation conducted on the training set
Yang et al [32]	MRI-anatomical-clinical features, morphological features	LR, SVM, RF, XGBoost	XGBoost	AUROC=0.88 (95% CI 0.74‐1.00), Accuracy=0.85, Sensitivity=0.90, Specificity=0.81	AUROC=0.82 (95% CI 0.68‐0.96), Accuracy=0.78, Sensitivity=0.81, Specificity=0.75	Data from 2 medical centers
Yin et al [33]	CT^ab^-based radiomics features, clinical factors	DNN, LR	DNN	AUC=0.92, Accuracy=0.75, Sensitivity=0.30, Specificity=0.83	Not reported	Random split (training set:test set=7:3), temporal split, class imbalance handling: SMOTE^aa^
Zheng et al [34]	Radiomics features, clinical factors, laboratory parameters	SVM	SVM	AUC=0.87 (95% CI 0.76‐0.94), Accuracy=0.76, Sensitivity=1, Specificity=0.65	AUC=0.81 (95% CI 0.72‐0.87), Accuracy=0.79, Sensitivity=0.87, Specificity=0.65	Center 1: partitioned into training and internal test sets. Center 2: designated as the external test set.
Zheng et al [35]	Tumor characteristics, surgical parameters, laboratory parameters	RF, MDN^ac^	RF	AUC=0.79 (95% CI 0.65‐0.93), Accuracy=0.82	Not reported	Random split (training set:test set=8:2), bootstrap
Zong et al [36]	Multiparametric MRI	DL	MS-3D-ResNet^ad^	AUC=0.87 (95% CI 0.86‐0.89), Accuracy=0.85, Sensitivity=0.86, Specificity=0.85	Not reported	Random split (training set:test set=7:3)
Li [37]	Clinical risk factors in obstetrics	LR, DT, KNN, BPNN^ae^, XGBoost, LGBM	LR	AUC=0.88 (95% CI 0.83‐0.92), Accuracy=0.77, Sensitivity=0.84, Specificity=0.67, PPV^af^=0.78, NPV^ag^=0.75	Not reported	5-fold cross-validation

a ML: machine learning.

b MRI: magnetic resonance imaging.

c DL: deep learning.

d XGBoost: extreme gradient boosting.

e VGG-16: visual geometry group - 16 layers.

f AUC: area under the curve.

g LR: logistic regression.

h Bayes: naïve Bayes.

i MLP: multilayer perceptron.

j DT: decision tree.

k KNN: k-nearest neighbors.

l RF: random forest.

m SVM: support vector machine.

n ECOG: eastern cooperative oncology group.

o DNN: deep neural network.

p MELD: model for end-stage liver disease.

q NN: neural network.

r AUROC: area under receiver operating characteristic curve.

s AUPR: area under the precision versus recall curve.

t LGB: light gradient boosting machine (LightGBM).

u CatB: categorical boosting (CatBoost).

v AdaB: adaptive boosting (AdaBoost).

w LSTM: long short-term memory.

x ADASYN: adaptive synthetic sampling.

y SGD: stochastic gradient descent.

z CNN: convolutional neural networks.

aa SMOTE: synthetic minority over-sampling technique.

ab CT: computed tomography.

ac MDN: mixture density network.

ad MS-3D-ResNet: multi-stream 3D residual network.

ae BPNN: back propagation neural network.

af PPV: positive predictive value.

ag NPV: negative predictive value.

### Model Validation Strategies

Although internal validation was widely applied (22/23, 96% [10,12,13,18,20-37]), its methodological rigor was insufficient (Table 4). Only half of the studies (12/23, 52% [10,12,13,18,20,22-27,33]) established an independent test set to evaluate final performance; even fewer used cross-validation (9/23, 39% [10,12,21,26,27,30,31,33,37]). External validation was notably lacking, implemented in only 6 studies (26%) [12,13,18,19,32,34]. Critically, among the limited external validations, model performance generally declined. For example, the model by Shi et al [13] dropped from an internal AUC of 0.85 to an external AUC of 0.80; when de Reus et al [19] independently validated the same model in a multinational, multicenter setting, the AUC further decreased to 0.63.

### Completeness of Performance Metric Reporting

There was substantial selective bias in the reporting of key performance metrics (Table 4). Discrimination metrics AUC were reported most frequently (19/23, 83% [10,12,13,18-20,23,24,26,27,29-37]), whereas reporting of essential classification metrics was incomplete: sensitivity (16/23, 70% [12,13,19,21,22,24,27-34,36,37]), specificity (14/23, 61% [12,19,21,22,24,27-29,31-34,36,37]). Reporting rates for precision (4/23, 17% [13,22,30,37]) and *F*_1_-score (4/23, 17% [12,13,19,30]) were very low. Furthermore, only 10/23 (43%) [13,18,19,25,26,28,32-34,36] of the studies reported model calibration (eg, calibration curves).

### Data Preprocessing and Interpretability

Reporting of data-preprocessing pipelines was seriously deficient (Table 5). In total, 11 studies (47%) [10,20,21,23,30-34,36,37] did not describe any method for handling missing data. Only 3 studies (13%) [13,24,33] reported strategies to address class imbalance (eg, using the synthetic minority oversampling technique [SMOTE]). The vast majority of studies neither applied nor reported any model interpretability analyses (eg, Shapley Additive Explanations [SHAP] and local interpretable model-agnostic explanations), rendering the models essentially “black-box.”

**Table 5.** Summary of data preprocessing methods in included studies (n=23).

Author	Missing data handling	Class imbalance handling	Data normalization or standardization
Akazawa and Hashimoto [10]	Not reported	Not reported	Not reported
Akazawa and Hashimoto [18]	Exclusion of cases with missing data	Not reported	Standardization of all radiomic features
Chen et al [12]	Multiple imputation using MICE^a^ package	Not reported	Standardization: numerical variables were standardized
de Reus DC et al [19]	Multiple imputation combined with exclusion	Not reported	Not reported
Li et al [20]	Not reported	Not reported	Not reported
Liu et al [21]	Not reported	Not reported	Not reported
Mo et al [22]	Missing values were filled with 0	Not reported	Not reported
Park et al [23]	Not reported	Not reported	Not reported
Shi et al [13]	Median imputation	SMOTE^b^ Tomek	Not reported
Shi et al [24]	KNN^c^ imputation	ADASYN^d^	Not reported
Stehrer et al [25]	Exclusion if >25% missing; mean or mode imputation if <25%	Not reported	Not reported
Sun et al [26]	Exclusion of patients with missing key indicators	Not reported	Not reported
Wang [27]	Not reported	Not reported	Z-score normalization
Wakiya et al [28]	Not reported	Not reported	Not reported
Xu [29]	Exclusion of patients with missing key indicators	Not reported	MRI^e^ pixel values scaled to [0,1]
Xue et al [30]	Not reported	Not reported	Not reported
Yang et al [31]	Not reported	Not reported	Not reported
Yang et al [32]	Not reported	Not reported	Not reported
Yin et al [33]	Not reported	SMOTE	Not reported
Zheng et al [34]	Not reported	Not reported	Not reported
Zheng et al [35]	Not reported	Not reported	Standardization and normalization applied
Zong et al [36]	Not reported	Not reported	Not reported
Li [37]	Not reported	Not reported	Not reported

a MICE: multivariate imputation by chained equations.

b SMOTE: synthetic minority oversampling technique.

c KNN: k-nearest neighbors.

d ADASYN: adaptive synthetic sampling.

e MRI: magnetic resonance imaging.

### Risk-of-Bias Assessment of Included Studies

Based on a systematic evaluation using the PROBAST (Table 6), all included studies (23/23, 100% [10,12,13,18-37]) were judged to have an overall “high” risk of bias. High risk primarily stemmed from 2 domains—the “participants” domain (23/23, 100% [10,12,13,18-37], due to selection bias inherent in retrospective designs) and the “analysis” domain (20/23, 87% [10,13,18,20-25,27-37], mainly attributable to inconsistent data preprocessing and shortcomings in validation strategies).

**Table 6.** Risk of bias assessment of included models (n=23 studies).

Author	Participants	Predictors	Outcome	Analysis	Overall
Akazawa and Hashimoto [10]	High	Low	Unclear	High	High
Akazawa and Hashimoto [18]	High	Low	Unclear	High	High
Chen et al [12]	High	Unclear	Unclear	Low	High
de Reus DC et al [19]	High	Unclear	Unclear	Low	High
Li et al [20]	High	Low	Low	High	High
Liu et al [21]	High	Unclear	Unclear	High	High
Mo et al [22]	High	Unclear	Low	High	High
Park et al [23]	High	Low	Low	High	High
Shi et al [13]	High	Low	Low	High	High
Shi et al [24]	High	Unclear	Unclear	High	High
Stehrer et al [25]	High	Low	High	High	High
Sun et al [26]	High	Low	Low	Low	High
Wang [27]	High	High	Low	High	High
Wakiya et al [28]	High	High	High	High	High
Xu [29]	High	High	High	High	High
Xue et al [30]	High	High	High	High	High
Yang et al [31]	High	High	High	High	High
Yang et al [32]	High	Low	High	High	High
Yin et al [33]	High	Unclear	High	High	High
Zheng et al [34]	High	Low	Low	High	High
Zheng et al [35]	High	Low	Low	High	High
Zong et al [36]	High	Low	Low	High	High
Li [37]	High	High	High	High	High

## Discussion

### Principal Findings

This systematic scoping review synthesizes the current state of ML in predicting intraoperative bleeding in patients undergoing surgery. The results indicate that ML models demonstrate good discriminative ability (mean AUC 0.82, SD 0.008) and, in some scenarios, outperform traditional methods [10]. Multimodal data (eg, EHR combined with medical imaging) can further enhance predictive efficacy, aligning with the paradigm shift from “unimodal perception” to “multimodal cognition” [38]. However, the PROBAST assessment reveals a fundamental contradiction; despite significant technical potential, current studies exhibit a universally high risk of bias, particularly in the analysis domain (22/23, 87% of the included studies [10,13,18,20-25,27-37]). This raises serious concerns that the reported performance metrics are likely overestimated. Specifically, this systematic risk of overestimation stems from three interconnected methodological shortcomings: (1) selective reporting and optimization bias, whereby studies tend to report only the best-performing models and favorable metrics (eg, AUC) while omitting critical measures such as calibration; (2) inadequate internal validation strategies, characterized by reliance on simple data splitting without temporal validation, which may lead to overfitting and overly optimistic performance estimates; and (3) insufficient handling of critical data issues, like class imbalance and missing data, which can artificially inflate discrimination metrics. Collectively, these flaws indicate that the reported mean AUC of 0.82 (SD 0.008) likely reflects optimal laboratory performance under ideal conditions, rather than the true generalizability of the models to independent, prospectively collected clinical data. This view is corroborated by the commonly observed performance degradation in the limited external validations available, where models often exhibit significant drops in AUC when applied to independent cohorts [13,19]. Based on this, the subsequent discussion of this review will systematically focus on these three core aspects—the completeness of model performance reporting, the rigor of validation strategies, and the transparency of data preprocessing and interpretability.

First, there is severe selective bias in the reporting of model performance, which limits a comprehensive assessment of their clinical applicability. Current research is overly focused on reporting discrimination metrics (AUC reported in 19/23, 83% of studies [10,13,18,20-25,27-37]), while seriously neglecting calibration (reported in 10/23, 43% [13,18,19,25,26,28,32-34,36]) and key classification metrics (eg, precision and *F*_1_-score, reported in 4/23, 17% [12,13,19,30]). This bias obscures two core issues. First, the widespread absence of model calibration assessment undermines the clinical credibility of predicted probabilities. Calibration reflects the consistency between predicted probabilities and actual risks, serving as the direct basis for risk stratification [39]. However, only a minority of studies reported calibration results [13,18,19,25,26,28,32-34,36]. More critically, calibration performance is unstable and cannot be inferred from a high AUC. For example, one study [33] reported good internal calibration, whereas independent external validation [19] revealed significant miscalibration. This suggests that calibration must be independently evaluated, as its issues are often exposed during external validation. Furthermore, its absence in most studies casts doubt on the reliability of their “risk probability” outputs. Second, incomplete reporting of key classification metrics hinders the judgment of model utility. Precision is crucial for assessing alert efficiency and preventing alarm fatigue, yet its reporting is severely inadequate [13,19,21,36]. This makes it impossible to quantify the model’s false-positive risk. For instance, a model [12] reported high sensitivity (eg, identifying most true bleeding events) but lower specificity, implying a higher number of false-positive alerts. Without reporting precision, the accuracy of these alerts cannot be quantified, making it difficult to assess whether this high-sensitivity strategy would lead to “alert fatigue” in practice. Conversely, the model developed by Xue et al [30] achieved high accuracy (eg, most of its alerts are true), but its sensitivity might be low, potentially missing a considerable proportion of true bleeding events, which could increase the risk of clinical under-diagnosis. The systematic absence of these key metrics makes it challenging to evaluate model robustness across different clinical decision thresholds. Therefore, future research must strictly adhere to reporting guidelines, such as Transparent Reporting of a Multivariable Prediction Model for Individual Prognosis or Diagnosis (TRIPOD) [40] and comprehensively present calibration and classification metrics to bridge the gap between technical development and clinical practice.

Second, model validation strategies generally lack rigor. The widespread absence of external validation, in particular, weakens the reliability of their generalizability assessment. This review found that although over half of the studies (12/23, 52% [10,12,13,18,20,22-27,33]) established an independent test set, their internal validation mostly relied on simple data splitting, with only one study [33] using the more robust temporal validation method. This overreliance on simple hold-out methods, coupled with limited adoption of methods such as cross-validation, may lead to optimistic performance estimates. More critically, external validation is severely lacking (only 6/23, 26% [12,13,18,19,32,34]), and performance degradation is commonly observed in implemented validations. This directly reveals the limited generalizability of models developed on homogeneous data. For example, the model by Shi et al [13] experienced a decrease in AUC from 0.85 in internal validation to 0.63 during multinational, multicenter external validation [19]. Models by Yang et al [32] and Zheng et al [34] showed similar trends in external performance decline. A notable exception is the model by Chen et al [12], which was built on large-scale multicenter data and showed improved performance in external validation, suggesting that an appropriate study design can enhance generalizability. In summary, the generalizability of existing models has not been sufficiently or rigorously validated. To further confirm the effectiveness and broad applicability of models in real-world settings, future research must incorporate prospective design, temporal validation, and multicenter external validation as key components of model evaluation.

Furthermore, insufficient transparency in data preprocessing and the widespread lack of model interpretability constitute another systemic methodological defect hindering research reproducibility and clinical translation. This review found that over 40% (11/23) of studies [10,20,21,23,30-34,36,37] did not report methods for handling missing data, and only 13% (3/23) [13,24,33] addressed class imbalance. The reporting of data preprocessing steps is severely deficient and nonstandard (eg, failing to clearly describe key procedures such as handling missing values and normalization [10,19-22,26,27,29-31]), thereby directly compromising model robustness and reproducibility. Although a few studies adopted more rigorous methods (eg, multiple imputation [12,18], SMOTE [33], or adaptive synthetic sampling [24]), simpler strategies that may introduce bias (eg, direct case deletion [17,25,28]) remain common. This lack of transparency makes interstudy comparison and independent replication exceptionally difficult and may partly explain the performance decline observed for some models during external validation [19]. Concurrently, model interpretability analysis is far from standard practice. The vast majority of studies lack any explanatory analysis (eg, SHAP values and feature importance), rendering them “black boxes” that clinical decision-makers find difficult to trust. Although a few studies have attempted to apply interpretability techniques, such as SHAP values or feature importance rankings [19,22], to identify key risk features and enhance transparency, this has not become routine. Therefore, future research must be committed to promoting the standardized reporting of data preprocessing workflows and deeply integrating interpretability analysis throughout the entire model development and validation process, which is a key prerequisite for building trustworthy and clinically usable prediction tools.

### Future Research Directions

Based on the findings of this review, to promote the transition of prediction models from “technically feasible” to “clinically applicable,” future research should focus on four core directions. First, promote rigorous validation and generalizability assessment. Model development must move beyond retrospective single-center designs, collect data through multicenter prospective studies, and use temporal validation and independent external validation as cornerstones of evaluation to rigorously test their robustness. Second, improve performance reporting and clinical utility evaluation. Research must strictly adhere to reporting guidelines, such as TRIPOD, and fully present performance metrics. Furthermore, methods such as decision curve analysis should be actively adopted to quantify the clinical net benefit of models across different decision thresholds, aligning evaluation with real-world decision-making scenarios. Third, standardize data processing and enhance model interpretability. Detailed reporting of data preprocessing workflows, along with the adoption of advanced methods for handling missing values and class imbalance, should become standard practice. Simultaneously, interpretability techniques, such as SHAP, should be integrated into the development pipeline as essential components to elucidate risk mechanisms and build clinical trust. Finally, explore clinical integration pathways and evaluate real-world impact. Current research in the field mostly remains at the stage of model development and technical validation, and its potential clinical value has not yet been substantiated. Specifically, building on preliminary evidence, future research should be dedicated to deepening and validating the following key translational aspects. First, promote the prospective application and effect evaluation of prediction models to guide preoperative blood preparation. Although existing models show potential to optimize blood preparation strategies [41,42], their impact on resource conservation and team response efficiency after integration into actual workflows remains to be confirmed by prospective studies. Second, expand the generalizability and clinical integration of real-time alert models. Although some studies have successfully developed real-time prediction models for intraoperative massive transfusion and demonstrated excellent performance [43], their generalizability across different surgical types and medical centers, as well as their actual alert efficacy and clinical acceptance after integration into anesthesia monitoring systems, requires further validation. Finally, and most challengingly, evaluate the improvement effect of model-based clinical decisions on patient hard endpoints through prospective interventional trials. Existing observational studies suggest that transfusion is associated with worse outcomes and higher costs [44]. Future well-designed studies are needed to confirm whether effective prediction-intervention strategies can ultimately achieve comprehensive benefits—such as reducing unnecessary transfusions and timely management of major bleeding—thereby lowering complications, improving patient prognosis, and saving medical costs.

### Limitations

The limitations of this review primarily stem from the methodological quality of the included original studies. First, the search strategy may not have captured all relevant literature, posing a risk of omission. More critically, the widespread retrospective design and high risk of bias in the current field necessitate cautious interpretation regarding the true performance and generalizability of the evaluated models.

### Conclusion

This scoping review indicates that research on ML for predicting intraoperative bleeding is growing rapidly in quantity, but the quality of studies has not improved correspondingly, constituting the main obstacle to clinical translation. Existing models are generally built on retrospective data and suffer from core methodological flaws, including a high risk of bias, a severe lack of external validation, and incomplete reporting of key performance metrics. Therefore, the clinical applicability and reliability of current models are far from established. To achieve the leap from methodological exploration to clinical utility, future research must meet higher standards—prioritize prospective design, enforce independent and multicenter external validation, strictly adhere to standardized reporting guidelines such as TRIPOD, and strive to explore effective pathways for integrating models into perioperative workflows.

## Supplementary material

10.2196/80930 Checklist 1 PRISMA checklist.

## Abbreviations

AUC: area under the curve
CHARMS: Critical Appraisal and Data Extraction for Systematic Reviews of Prediction Modeling studies
CNKI: China National Knowledge Infrastructure
EHR: electronic health record
ML: machine learning
MRI: magnetic resonance imaging
PICOS: Population, Intervention, Comparator, Outcome, and Study design
PRISMA-ScR: Preferred Reporting Items for Systematic Reviews and Meta-Analyses extension for Scoping Reviews
PROBAST: Prediction model Risk of Bias Assessment Tool
SHAP: Shapley Additive Explanations
SMOTE: synthetic minority over-sampling technique
TRIPOD: Transparent Reporting of a Multivariable Prediction Model for Individual Prognosis or Diagnosis
VIP: China Science and Technology Journal Database
